# ﻿Two new species of *Eccoptopterus* Motschulsky, 1863 ambrosia beetle from Taiwan and Thailand (Coleoptera, Curculionidae, Scolytinae, Xyleborini)

**DOI:** 10.3897/zookeys.1217.129707

**Published:** 2024-11-05

**Authors:** Wisut Sittichaya, Ching-Shan Lin, Sarah M. Smith, Chaninan Pornsuriya, Anthony I. Cognato

**Affiliations:** 1 Agricultural Innovation and Management Division, Faculty of Natural Resources, Prince of Songkla University, Songkhla, 90110, Thailand Prince of Songkla University Songkhla Thailand; 2 Department of Entomology, National Taiwan University, Taipei 10617, Taiwan National Taiwan University Taipei Taiwan; 3 Department of Entomology, Michigan State University, 288 Farm Lane, room 243, East Lansing, MI 48824, USA Michigan State University East Lansing United States of America

**Keywords:** Ambrosia beetle, molecular, new species, Taiwan, taxonomy, Thailand, xyleborine

## Abstract

Two xyleborine ambrosia beetles, *Eccoptopterusformosanus***sp. nov**. and *E.intermedius***sp. nov.** are described from Taiwan and Thailand, respectively, based on DNA sequences (COI and CAD) and morphological characteristics. A key to the *Eccoptopterus* species of Southeast Asia is provided.

## ﻿Introduction

*Eccoptopterus* Motschulsky, 1863 is one of the earliest described genera of xyleborine ambrosia beetles (Coleoptera: Curculionidae: Scolytinae). The Russian entomologist, Victor Ivanovich Motschulsky, erected the name for his monotypic genus and new species, *Eccoptopterussexspinosus* Motschulsky, 1863, described from Burma (now Myanmar) (Motschulsky 1863) which he classified under Hylesinidae. Eichhoff (1876) later moved the genus to Xyleborini and Schedl (1963) synonymized the type species, *E.sexspinosus*, with *Scolytusspinosus* Olivier, 1800. Two other synonymous genera were later published, *Platydactylus* Eichhoff, 1886 (preoccupied by Goldfuss 1820) and its new name *Eurydactylus* Hagedorn, 1909.

Fourteen species and subspecies have been described, of which four are currently recognized: *E.drescheri* Eggers, 1940, *E.limbus* Sampson, 1911, *E.spinosus* (Olivier, 1800), *E.tarsalis* Schedl, 1936. *Eccoptopterus* is easily distinguished by the autapomorphic enlarged metatibiae and metatarsi (Hulcr et al. 2007). However, *Eccoptopterus* species are noted for their exceptional morphological variation. This continuum of variation is especially apparent with specimens collected at different altitudes and is not correlated with geographic origin (Hulcr and Cognato 2013). *Eccoptopterusspinosus* has been previously reported to be a species complex (Cognato et al. 2011; Smith et al. 2020) in need of further study. Other xyleborine species complexes have been delimited using a combination of morphological characteristics and DNA sequence data (e.g Gomez et al. 2018; Smith et al. 2020; Smith and Cognato 2022; Smith et al. 2022). Based on specimens collected as part of WS’s survey of Thai xyleborine ambrosia beetles (Sittichaya et al. 2021) and CSL’s collecting in Taiwan, we discovered variation in *Eccoptopterus* specimens which suggested potential new species. To test the hypothesis that these new forms represent distinct species, morphological and molecular characters were investigated.

## ﻿Materials and methods

### ﻿Insect collection, imaging and terminology

Specimens of a putative new species from Thailand were collected from several provinces (Chiang Mai, Lamphun, Tak, Ubon Ratchathani) between 01.i.2019–31.xii.2020 using ethanol baited traps and fallen branches. The specimens of a putative new species from Taiwan were collected from fallen branches and logs from May 2016 to October 2023. These specimens were then compared with the type specimens, images of type specimens or by examining the original descriptions (Table 1). Photographs were taken with a Canon 5D and 50D digital cameras with a Canon MP-E 65 mm Macro Photo Lens (Canon, Tokyo, Japan) and StackShot-Macrorail (Cognisys Inc, Michigan, USA). The photos were then combined with Helicon Focus ver. 6.8.0. (Helicon Soft, Ukraine); all photos were improved with Adobe Photoshop CS6 (Adobe Systems, California, USA). The antennal and pronotum types and characters follow those proposed by Hulcr et al. (2007) and subsequently elaborated on by Smith et al. (2020).

**Table 1.** List of *Eccoptopterus* types, repository and method of examination.

Species	Synonym	Type and repository	Method of examination
*Eccoptopterusdrescheri* Eggers, 1940	–	Cotype (NHMW)	SMS, AIC examined
*Eccoptopteruslimbus* Sampson, 1911	–	Holotype (NHMUK)	SMS, AIC examined
*Eccoptopteruslimbus* Sampson, 1911	*Xyleborussquamulosusauratus* Eggers, 1923	Lectotype (NMNH)	Images; USNMENT_01547121
*Eccoptopteruslimbus* Sampson, 1911	*Xyleborussquamulosusduplicatus* Eggers, 1923	Lectotype (NMNH)	Images; USNMENT_01547119
*Eccoptopteruslimbus* Sampson, 1911	*Xyleborussquamulosus* Eggers, 1923	Lectotype (NMNH)	Images; USNMENT_01547120
*Eccoptopterusspinosus* (Olivier, 1800)	–	Holotype (MNHN)	Type not located (Hulcr and Cognato 2013)
*Eccoptopterusspinosus* (Olivier, 1800)	*Platydactylusgracilipes* Eichhoff, 1886	Syntypes (UHZM)	Types destroyed (Wood and Bright 1992)
*Eccoptopterusspinosus* (Olivier, 1800)	*Xyleborusabnormis* Eichhoff, 1869	Syntypes (UHZM)	Types destroyed (Wood and Bright 1992)
*Eccoptopterusspinosus* (Olivier, 1795)	*Xylebrousmultispinous* Hagedorn, 1908	Syntypes (MFNB)	Original description
*Eccoptopterusspinosus* (Olivier, 1800)	*Eccoptopterussagittarius* Schedl, 1939	Paratypes (NMNH)	Examined by SMS
*Eccoptopterusspinosus* (Olivier, 1800)	*Eccoptopterussexspinosuspluridentatus*	Lectotype (NHMW)	SMS, AIC examined
*Eccoptopterusspinosus* (Olivier, 1800)	*Eccoptopteruseccoptopterus* Schedl, 1951	Lectotype (NHMW)	SMS, AIC examined
*Eccoptopterusspinosus* (Olivier, 1800)	*Eccoptopteruscollaris* Eggers, 1923	Lectotype (NMNH)	WST examined, Images; USNMENT_01356999
*Eccoptopterusspinosus* (Olivier, 1800)	*Eccoptopterussexspinosus* Motschulsky, 1863	Syntypes (ZMMU)	Original description
*Eccoptopterustarsalis* Schedl, 1936	–	Holotype (NHMW)	SMS, AIC examined

### ﻿Abbreviations and terminology

**CSL** Private collection of Ching Shan Lin, Taichung, Taiwan;

**MFNB** Museum für Naturkunde, Berlin, Germany;

**MNHN**Muséum national d’Histoire naturelle, Paris, France;

**MSUC** Albert J. Cook Arthropod Research Collection, Michigan State University, East Lansing, USA;

**NHMW**Naturhistorisches Museum Wien, Austria;

**NMNS**National Museum of Natural Science, Taichung, Taiwan;

**NMNH**National Museum of Natural History, Smithsonian Institution, Washington, D.C., USA;

**NHMUK**Natural History Museum, London, UK;

**NTU** National Taiwan University Insect Museum, Taipei, Taiwan;

**THNHM** Natural History Museum of the National Science Museum, Pathumthani, Thailand;

**UHZM** Universität Hamburg – Zoological Museum, Hamburg, Germany;

**WSTC** Private collection of Wisut Sittichaya, Songkhla, Thailand;

**ZMMU**Zoological Museum at Moscow State University, Moscow, Russia;

**Major spines** are large, regularly present in homologous positions on declivital margin; one pair for *E.limbus* at summit, and three pairs for *E.spinosus* on declivital summit, middle and apex of declivity.

**Minor spines** are smaller and irregularly present in some positions.

### ﻿DNA extraction and phylogenetic analysis

#### ﻿Extraction and analysis

Two specimens of an unidentified *Eccoptopterus* morphospecies (SWE01, 02) from Thailand and a specimen of another *Eccoptopterus* morphospecies from Taiwan (SWE02T) were chosen for DNA extraction. The head and pronotum of each specimen were removed and placed in 1.5 ml microfuge tube. The genomic DNA from each specimen was extracted using DNEasy Blood and Tissue Kit (Qiagen Ltd., Hilden, Germany) according to the manufacturer’s protocol. PCR amplification of partial cytochrome *c* oxidase subunit I (COI) mtDNA gene and carbamoyl-phosphate synthetase 2, aspartate transcarbamylase, and dihydroorotase (CAD) was conducted by using primer pair COL6/COH6 (Schubart 2009) for COI and apCADfor4/apCADrevlmod (Danforth et al. 2006) for CAD. The PCR reaction mixtures contained DNA template, 10 *p*mol of each primer, 5x HOT FIREPol^®^ Blend Master Mix (Thistle Scientific Ltd, Scotland) and distilled water (DW) in 25 μl tube. PCR was performed in a BIO-RAD T100^TM^ Thermal Cycler (Hercules, CA, USA) and the PCR conditions for COI were 13 min at 95 °C, followed by 40 cycles of 95 °C for 20 sec, 55 °C for 30 sec, 72 °C for 1 min, and the final extension at 72 °C for 5 min. The annealing temperatures differed for the CAD gene with the optimum at 59 °C. PCR products were visualized by agarose gel electrophoresis and sequenced in both directions by Macrogen, Inc. (Seoul, South Korea).

Forward and reverse DNA sequences were aligned, edited and merged using MEGA X software (Kumar et al. 2018). The generated sequences were submitted to GenBank (http://www.ncbi.nlm.nih.gov) under accession numbers LC716017 and LC716018 for *COI* and LC815915, LC716015 and LC716016 for CAD sequences (Table 2). The sequences in this study were compared with sequences of *Eccoptopterus* species retrieved from GenBank. COI and CAD sequence data were concatenated, aligned with MEGA X software using ClustalW algorithm and manually adjusted as necessary. Phylogenetic tree estimation for each alignment was performed using maximum parsimony (MP), maximum likelihood (ML), and Bayesian inference (BI). The MP tree was obtained using the heuristic search option with 1000 random additions of sequences and tree bisection and reconnection (TBR) as the branch-swapping algorithm using MEGA X. The ML tree was constructed using MEGA X using the General Time Reversible (GTR) nucleotide subsitution model for tree inference and 1000 bootstrap replicates. The Bayesian tree was generated using MrBayes ver. 3.2.7a (Ronquist et al. 2012). Markov chain Monte Carlo (MCMC) runs were performed for 1,000,000 generations and sampled every 100 generations. The initial 25% of generations were discarded as burn-in, and the remaining trees were used to calculate the Bayesian inference posterior probability (BPP) values. Phylogenetic trees were visualized by using FigTree ver. 1.4.4 (http://tree.bio.ed.ac.uk/software/figtree/). DNA percent difference was measured as pairwise uncorrected “p” distance.

**Table 2.** *Eccoptopterus* species and isolates used in the phylogenetic analyses, with GenBank accession numbers.

Species	Specimen/voucher	Location	GenBank accession	
			COI	CAD
*Anisandruscristatus*	SAX290	Vietnam: Cao Bang	MN619841	MN620134
*Eccoptopteruslimbus*	Ecclim_258	Borneo	HM064081	HM064261
*E.spinosus*	SAX150	Vietnam: Dong Nai	MN619920	MN620195
*E.formosanus* sp. nov.	SAX64	Taiwan	MN619919	N/A
*E.spinosus*	SAX63	Indonesia: Java	MN619918	MN620194
*E.spinosus*	Eccspi	Papua New Guinea	HM064082	HM064262
*E.spinosus* (*E.gracilipes*)	Eccgra	Papua New Guinea	HM064080	HM064260
***E.formosanus* sp. nov.**	SWE02T	Taiwan	LC815914	LC815915
***E.intermedius* sp. nov.**	SWE01	Thailand: Ubon Ratchathani	LC716017	LC716015
***E.intermedius* sp. nov.**	SWE02	Thailand: Chiang Mai	LC716018	LC716016

Specimens sequenced in this study are indicated in **bold**. N/A: Not available.

#### ﻿Species concept

We consider Xyleborini species as hypotheses of evolutionary independent lineages (Hey 2006). Monophyly of individuals, inferred from a phylogeny, demonstrates an evolutionary lineage and suggests the recognition of a species. Species recognition is based on monophyly of individuals with unique diagnostic characters similarly observed with other recognized species and a percent nucleotide difference near the threshold established for Xyleborini of >10% COI and > 2% CAD pairwise uncorrected “p” distance between sister clades (Cognato et al. 2020).

## ﻿Results

### ﻿Molecular evidence

The COI and CAD sequences compared between *Eccoptopterus* spp. demonstrated clear differences and confirmed the new species status of both species from Thailand and Taiwan. The sequences of SWE01 and SWE02 differ from *E.spinosus* and *E.limbus* in COI between 14.85−15.36% and in CAD between 5.64−7.65%. Similar values were found between the new species from Thailand and Taiwan 15.32−15.77% for COI and 3.88−4.0% for CAD. The percentages of both genes for both species exceed the suggested species boundary of 10% and 2% (Table 3).

**Table 3.** DNA percent difference of *E.formosanus* sp. nov. (SWE02T) and *E.intermedius* sp. nov. (SWE01-02) to species in the NCBI (National Center for Biotechnology Information) database.

Specimen	Gene	Species with the most related sequence	GenBank number	Difference (%)
SWE02T	COI	*Eccoptopterusspinosus* Java	MN619918	15.91
		*Eccoptopterusformosanus* Taiwan SAX64	MN619919	00.00
		*Eccoptopterusspinosus* VN Cat	MN619920	15.30
		*Eccoptopterusspinosus* PNG1	HM064082	14.88
		*Eccoptopterusspinosus* Eccgra	HM064080	17.78
		*E.intermedius* sp. nov. (SWE01)	LC716017	15.78
		*E.intermedius* sp. nov. (SWE02)	LC716018	15.33
		*Eccoptopteruslimbus* Borneo	HM064081	15.58
	CAD	*Eccoptopterusspinosus* Java	MN620194	7.94
		*Eccoptopterusspinosus* Taiwan SAX64	N/A	N/A
		*Eccoptopterusspinosus* VN Cat	MN620195	8.81
		*Eccoptopterusspinosus* PNG1	HM064262	6.88
		*Eccoptopterusspinosus* Eccgra	HM064260	6.83
		*E.intermedius* sp. nov. (SWE01)	LC716015	3.88
		*E.intermedius* sp. nov. (SWE02)	LC716016	4.07
		*Eccoptopteruslimbus* Borneo	HM064261	6.11
SWE01	COI	*Eccoptopterusspinosus* SAX63	MN619918	15.36
		*Eccoptopterusgracilipes* (*E.spinosus*)	HM064080	14.85
		*Eccoptopterusspinosus*	HM064082	14.90
		*Eccoptopterus* sp. Ecc1487_270	MN619915	15.04
	CAD	*Eccoptopterus* sp. 329	HM064259	6.36
		*Eccoptopteruslimbus* 258	HM064261	6.55
		*Eccoptopterusgracilipes* 12341 (*E.spinosus*)	MK098872	6.26
		*Eccoptopterusspinosus* SAX331	MN620196	7.22
		*Eccoptopterusgracilipes* (*E.spinosus*) Eccgra	HM064260	5.69
		*Eccoptopterusspinosus* SAX63	MN620194	7.40
		*Eccoptopterusspinosus* Eccspi	HM064262	5.61
		*Eccoptopterusspinosus* SAX150	MN620195	7.22
SWE02	COI	*Eccoptopterusspinosus*	HM064082	15.36
	CAD	*Eccoptopterus* sp. 329	HM064259	6.57
		*Eccoptopteruslimbus* 258	HM064261	6.77
		*Eccoptopterusgracilipes* 12341 (*E.spinosus*)	MK098872	6.22
		*Eccoptopterusspinosus* SAX331	MN620196	7.61
		*Eccoptopterusgracilipes* (*E.spinosus*) Eccgra	HM064260	5.64
		*Eccoptopterusspinosus* SAX63	MN620194	7.57
		*Eccoptopterusspinosus* Eccspi	HM064262	5.87
		*Eccoptopterusspinosus* SAX150	MN620195	7.65

**Figure 1.**
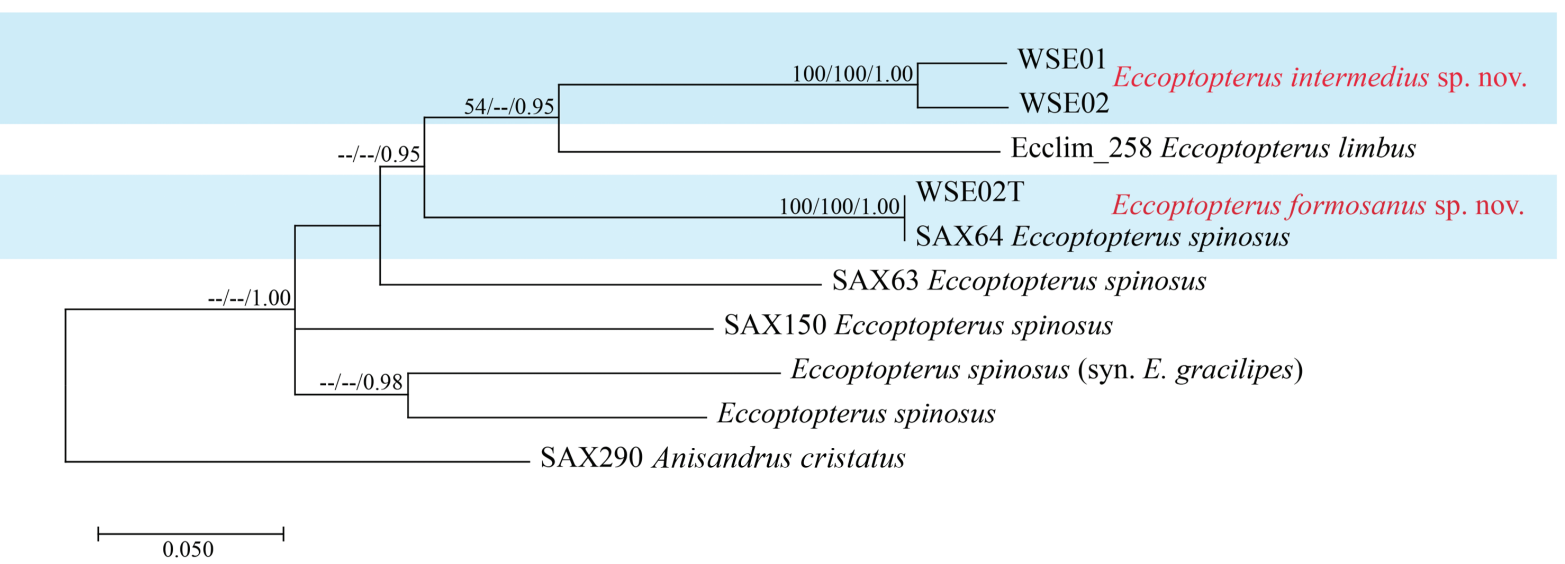
Phylogenetic tree generated by maximum likelihood analysis based on the combined sequences of COI and CAD sequence data of *Eccoptopterus*. Bootstrap values for maximum likelihood (ML) and maximum parsimony (MP) equal to or greater than 70% and Bayesian posterior probabilities (BPP) equal or greater than 0.95 are placed above the branches, respectively. The new species are indicated in blue area. The tree is rooted to *Anisandruscristatus*.

The maximum likelihood (ML), maximum parsimony (MP) and Bayesian inference (BI) for phylogenetic analyses of combined sequence COI (585 characters) and CAD (376 characters) resulted in trees with similar topologies. Phylogenetic results (Fig. 1) showed that two specimens (SWE01 and SWE02), representing *E.intermedius*, clustered together and SWE02T, representing *E.formosanus* had an identical sequence to a specimen previously identified as *E.spinosus* (SAX64) from Taiwan and included in the study of Cognato et al. (2020). Each species formed a distinct lineage within *Eccoptopterus* and were recovered as sister to *E.limbus* but each can be recognized as a phylogenetically distinct species (Fig. 1).

### ﻿Taxonomic treatment

#### ﻿ Eccoptopterus

Taxon classificationAnimaliaColeopteraCurculionidae

Motschulsky, 1863

7AA144DC-FF02-5139-857A-6397E432DF12

Eccoptopterus Motschulsky, 1863: 515.
Platydactylus Eichhoff, 1886: 25. Preoccupied by Goldfuss, 1820.
Eurydactylus Hagedorn, 1909: 733. (new name for Platydactylus Eichhoff, 1866 nec. Goldfuss 1820; preoccupied by Laferté-Sénectère, 1851). Synonymy: Hagedorn 1910: 110.

##### Type species.

*Eccoptopterussexspinosus* Motschulsky, 1863 = *Scolytusspinosus* Olivier, 1800.

##### Diagnosis.

1.70−5.90 mm, stout, 1.94−2.3× as long as wide; pronotum short and round, robust, broader and larger than elytra; pronotal anterior margin armed with a pair of prominently protruding denticles; pronotal base bearing a dense tuft of setae; elytra short, excavated, with denticles around its margins; declivity impressed, the impressed areas extending nearly to elytral base; metatibiae conspicuously enlarged and flattened.

#### ﻿ Eccoptopterus formosanus

Taxon classificationAnimaliaColeopteraCurculionidae

Lin, Sittichaya & Smith sp. nov.

DBA136A0-B71D-5D53-9C00-E4F804A32E70

https://zoobank.org/DFC6F8B0-22B2-4983-B744-411097C5F087

Fig. 2

##### Type material.

***Holotype***: • female, Taiwan, Nantou county, Ren’ai Township; 24°0'0.3675"N, 121°0'34.4817"E; 969 m a.s.l.; a diameter 4.5 cm branch of *Sapiumdiscolor* (Euphorbiaceae); 02.iv.23, (C. S. Lin) (NMNS). ***Paratypes***: • male, same as holotype (1, NMNS), female, (3, MSUC), 1 female, 1 male (2 NTU), 1 male, 14 females (15, CSL) • female, Nantou County (Lugu Township); 23°45'0.31"N, 120°48'59.99"E; 720 m a.s.l.; a diameter 5.2 cm branch of *Elaeocarpussylvestris* (Elaeocarpaceae); 022.vi.23, (C. S. Lin), 1 male, 4 females (5, WSTC), female (1, NMNH), (1, NHMUK).

##### Diagnosis.

**Female**, 2.56−2.64 mm long (mean = 2.61 mm; *N* = 4), 2.13−2.17× as long as wide (mean = 2.14×; *N* = 4). Medium body size, declivital armature composed of a pair of major spines on declivital summit and 2–4 minor denticles unevenly spaced on each lateral margin; protibiae slender, broadest at apical 1/3, outer margin armed with six or seven moderated socketed denticles; scutellum broadly linguiform; elytra tapering laterally.

##### Description.

**Figure 2.**
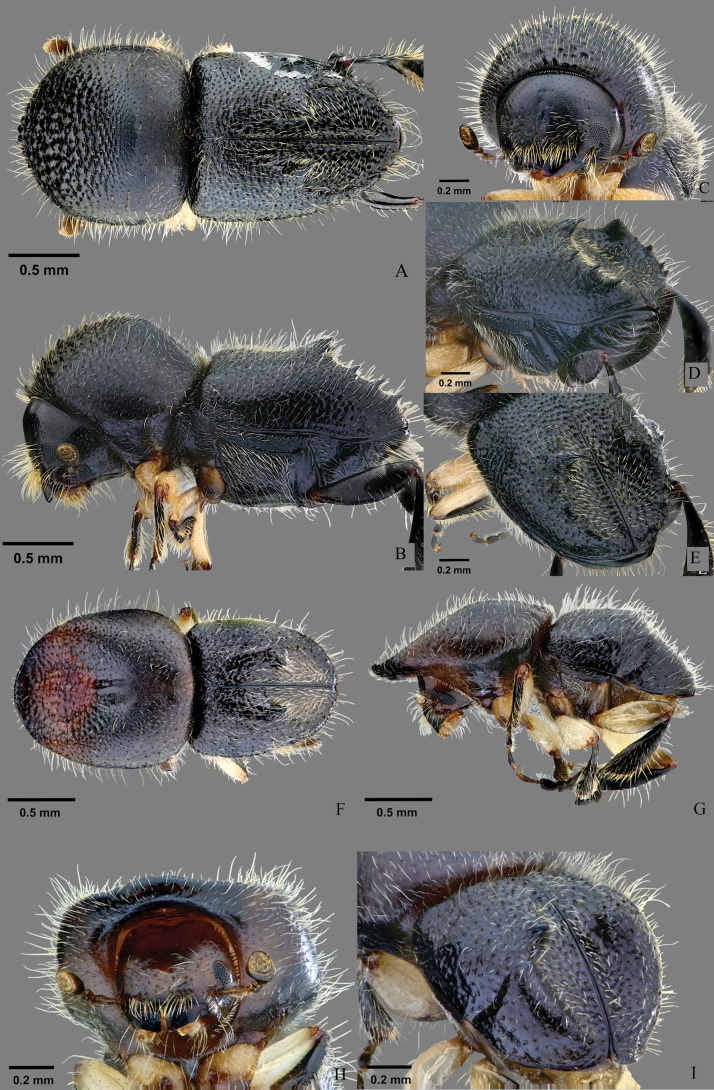
*Eccoptopterusformosanus* sp. nov. **A–E** holotype female **A** dorsal view **B** lateral view **C** frons **D** posterolateral view of abdomen **E** declivital face; **F–I** paratype male **F** dorsal view **G** lateral view **H** frons **I** posterolateral view of abdomen.

**Female** (Fig. 2A–E). Black brown, procoxae light brown, profemora and mesofemora paler brown, antennae, tibiae dark brown. ***Head***: epistoma entire, transverse, with a row of hair-like setae. Frons below upper margin of eye and above epistoma flat, flattened area broadly rounded, surface subshiny, finely reticulate, sparsely punctate, punctures bearing fine, yellowish-white, hair-like setae. Eye shallowly emarginate just above antennal insertion, upper portion slightly smaller than lower portion. Submentum triangular small, moderately impressed. Antennal scape long, normal thick, slightly longer than club (12:10). Pedicel as broad as scape, as long as funicle. Funicle 4-segmented, segment 1 shorter than pedicel. Club obliquely truncate, longer than wide (10:9), type 1, segment 1 corneous, occupying basal 1/4, margin carinate, concave, encircling anterior face, segment 2 and 3 soft, visible on anterior face only. ***Pronotum***: 0.97−1.00 (mean = 0.99, *N* = 4) × as long as wide, type 1 in dorsal view, lateral sides parallel to anterior middle, broadly rounded anteriorly; anterior margin with 4−6 serrations, median pair prominent; anterior slope strongly asperate, asperities densely spaced, rugose, lower and more transverse toward the summit; disc slightly convex, finely reticulate, dull, sparsely covered with fine punctures bearing fine short hair-like setae. Base with a tuft of short hair-like setae associated with mycangium. In lateral view short and tall, type 3, summit at middle, lateral margins obliquely costate. ***Elytra***: 1.14−1.17 (mean = 1.16, *N* = 4) × as long as wide, 1.10−1.17 (mean = 1.12, *N* = 4) × as long as pronotum. Scutellum comparatively moderately sized, narrow, linguiform, subshiny, attached on anterior slope of elytra less visible from above. Base shallowly bisinuate, with oblique edge, humeral angles rounded, lateral side tapering from humeral angle to apex. Disc short, basal area 1/4 of disc slightly convex, apical 3/4 impressed and connecting to declivital impression; disc punctate, punctures fine confused and setose, striae and interstriae hardly marked due to irregular punctures. Declivity sulcate, with a pair of major (largest) spines on declivital summit and 2–4 much smaller denticles on declivital margin; striae and interstriae punctate, punctures small and shallow, each bearing a short, semi-recumbent seta. ***Legs***: procoxae contiguous; prosternal coxal piece short, conical. Protibiae slender, broadest at middle; posterior face inflated, punctate, densely covered with long hair-like setae; outer margin armed with six or seven moderate socketed denticles. Meso- and metatibiae rounded, flat, mesotibiae armed with seven or eight smaller socketed denticles, metafemora and metatibiae enlarged, metatibiae without spines.

**Male.** (Fig. 2F–I). 2.18−2.30 mm (mean = 2.24 mm; *N* = 4) long, 1.81−2.04 (mean 1.95; *N* = 4) × as long as wide. Head reddish-brown, coxae light brown, femora paler brown, antennae, tibiae, pronotum and elytra dark brown in mature specimens, excepting impressed portion of pronotum is reddish-brown. ***Head***: somewhat margined laterally and concealed under the projection of pronotum. Epistoma entire, transverse, with a row of hair-like setae. Frons below upper margin of eye and above epistoma flat, the flattened area quadrate, surface subshiny, finely reticulate, sparsely shallow punctured, each bearing a short or long hair-like seta. Eye reduced, shallowly emarginate just above antennal insertion, upper portion slightly smaller than lower portion. Submentum triangular, small, slightly impressed. Antennal scape long, normal thick, slightly longer than club. Pedicel as broad as scape, as long as funicle. Funicle 4-segmented, segment 1 shorter than pedicel. Club obliquely truncate, longer than wide (7.5:5.5), type 1, segment 1 corneous, occupying basal 1/4, margin carinate, concave, encircling anterior face, segments 2 and 3 soft, visible on anterior face only. ***Pronotum***: 0.89−1.11 (mean = 0.99; *N* = 4) × as long as wide, type 1 in dorsal view, lateral sides subparallel to anterior middle, broadly rounded anteriorly; anterior margin unarmed; widely impressed on anterior slope, then gradually narrowing and slightly impressed toward disc, weakly asperate, asperities sparsely spaced, sub-rugose, becoming lower and more transverse toward the summit; slightly longitudinally impressed in middle of disc, finely reticulate, dull, sparsely covered with fine punctures bearing fine short hair-like setae. In lateral view long and tall, type 9, summit at apical 2/3, lateral margins obliquely costate. ***Elytra***: 1.00−1.05 (mean = 1.04; *N* = 4) × as long as wide, 0.80−1.00 (mean = 0.88; *N* = 4) × as long as pronotum. Scutellum small, linguiform, subshiny, attachment on anterior slope of elytra less visible from above. Base procurved, with oblique edge, humeral angles rounded, subparallel-sided in basal 1/2, then gradually incurved to broadly rounded apex. Disc short, punctate, punctures fine confused and setose, strial setae uniseriate with long, erect hair-like setae; interstrial setae uni- or biseriate with semi-recumbent hair-like setae; basal area 1/4 of disc slightly convex. Declivity somewhat steeply sloping, face weakly bisulcate, declivital face much lower than declivital margin, with a pair of major spines on declivital summit and 0–2 much smaller minor tubercles on declivital margin; striae and interstriae punctate, punctures small and shallow, each bearing a short, semi-recumbent setae. ***Legs***: procoxae narrowly separated; prosternal coxal piece short, conical. Protibiae slender, broadest at the middle; posterior face inflated, punctate, densely covered with long hair-like setae; outer margin armed with five or six moderate socketed denticles. Meso- and metatibiae rounded, flat, mesotibiae armed with six or seven smaller socketed denticles, metafemura and metatibiae enlarged, metatibiae without apical spine.

##### Etymology.

Formosa, the former name of Taiwan island, in reference to the collection locality of types. An adjective.

##### Distribution.

Taiwan (Nantou County).

##### Biology.

Bred from *Elaeocarpussylvestris* (Lour.) Poir. (Elaeocarpaceae), *Lithocarpushancei* (Benth.) Rehder, *Quercusglauca* Thunb. ex Murray (Fagaceae), *Sapiumdiscolor* Muell.-Arg. (Euphorbiaceae), *Tremaorientale* (L.) Blume (Cannabaceae) with a diameter of about 4.8–6.2 cm in Taiwan. The radial entrance gallery leads to several branches in various planes without enlarged brood chambers (C. S. Lin pers. obs.).

#### ﻿ Eccoptopterus intermedius

Taxon classificationAnimaliaColeopteraCurculionidae

Sittichaya, Lin & Smith sp. nov.

F2E0A6A1-8C5F-5F87-8C6C-BB465C1AB33D

https://zoobank.org/BCEE85CB-6BE4-412A-B1B7-E3096D103600

Fig. 3

##### Type material.

***Holotype***, • female, Thailand, Tak Province, 17°40'17.7"N, 97°51'04.2"E; 600 msl; semiagricultural area, ex. small branch of unknown tree; 08.ix.19, (W. Sittichaya) (NHMW); ***Paratypes***: • females, Ubon Ratchathani Province, Pha Taem National Park, 15°37'21.9"N, 105°36'34.7"E; 420 m a.s.l.; dry dipterocarp rainforest, ethanol baited trap; 01.v.2019, (W. Sittichaya) • Tak Province, 17°40'17.7"N, 97°51'04.2"E; 600 m a.s.l.; semiagricultural area, ex. Small branch of unknown tree; 08.ix.19 (1), (W. Sittichaya), (1 THNHM) • Lamphun Province, Maeping National Park, 17°33'29.6"N, 98°52'46.0"E; 600 m a.s.l.; Dry Dipterocarp forest, ethanol baited trap; 01.ii.19 (1), 01.iii.19 (1), 01.v.19 (1) (all W. Sittichaya) (3 WSTC) • Chiang Mai Province, Chiang Dao Wildlife Sanctuary, 17°33'29.6"N, 98°52'46.0"E; 600 m a.s.l.; mixed deciduous forest, ethanol baited trap; 01.vi.19 (W. Sittichaya) (1 MSUC).

**Figure 3.**
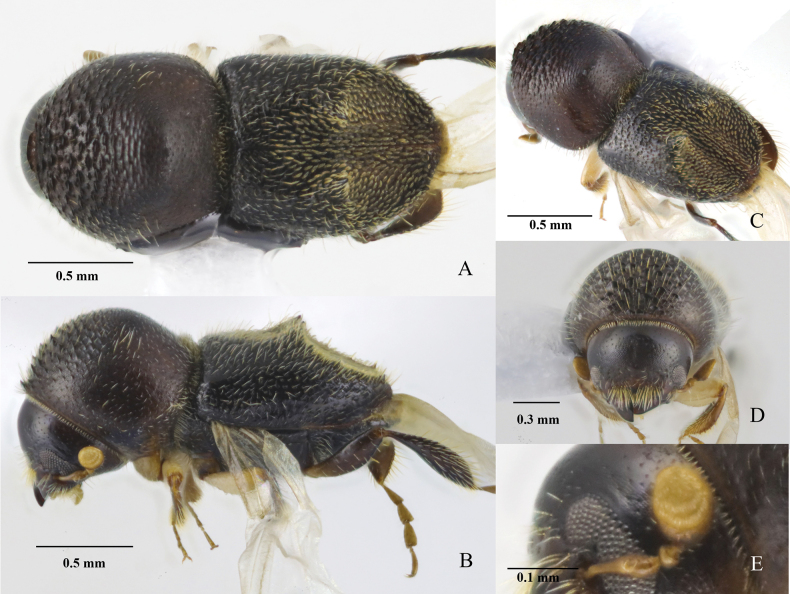
*Eccoptopterusintermedius* sp. nov. Holotype, female, **A** dorsal view **B** lateral view **C** posterolateral view **D** frons **E** antenna.

##### Diagnosis.

**Female**, 1.70−1.90 mm long (mean = 1.80 mm; *N* = 6), 2.03−2.38× as long as wide (mean = 2.13×; *N* = 6). Small body size, declivital armature composed of a pair of major spines at interstriae 3 on declivital summit and four minor spines unevenly spaced on each lateral margin, declivity covered with flattened scale-like setae; protibiae slender, broadest at apical 1/3, outer margin armed with four or five moderated socketed denticles, elytra tapering laterally.

##### Description.

**Female**. Body brown, dark brown to black, impressed portion of elytral disc and declivital face paler and bearing grayish-brown scale-like setae; antennae, prolegs, middle legs and associated coxae paler brown, hind legs dark brown to black. ***Head***: epistoma complete, margin bisinuated, with a row of hair-like setae. Frons below upper margin of eye and above epistoma impressed, without raised median line, surface reticulate, subshiny, sparsely covered with fine long setae, setal insertion shallowly punctate. Frons below upper portion of the eye slightly convex. Eye shallowly emarginate just above antennal insertion, upper portion slightly smaller than lower part. Submentum triangular small, shallowly impressed. Antennal scape long, normal thick, slightly longer than club (9:8). Pedicel as broad as scape, as long as funicle. Funicle 4-segmented, segment 1 shorter than pedicel. Club obliquely truncate, longer than wide (8:6.5), type 1, segment 1 corneous, occupying basal 1/4, margin carinate, concave, encircling in anterior face, segment 2 and 3 soft, visible on anterior face only. ***Pronotum***: 0.93−0.97 (mean = 0.95, *N* = 6) × as long as wide, round shorter than long, type 1 in dorsal view, lateral sides parallel to anterior middle, broadly rounded anteriorly; anterior margin with 2−4 serrations, median pair prominent; anterior slope strongly asperate, asperities densely spaced, rugose, becoming lower and more transverse toward the summit; disc slightly convex, finely alutaceous, dull, sparsely covered with fine punctures bearing fine short hair-like setae. Base with a tuft of short hair-like setae associated with mycangium. In lateral view short and tall, type 3, summit at middle, lateral margins obliquely costate. ***Elytra***: 1.12−1.18 (mean = 1.15, *N* = 6) × as long as wide, 1.07−1.19 (mean = 1.15, *N* = 6) × as long as pronotum. Scutellum comparatively moderately sized, narrow, linguiform, finely punctate, subshiny, attachment on anterior slope of elytra less visible from above. Base shallowly bisinuate, with oblique edge, humeral angles rounded, lateral side tapering from humeral angle to apex. Disc short, basal area ¼ of disc slightly convex, apical 3/4 impressed and connecting to declivital impression; disc punctate, punctures fine confused and setose, strial setae uniseriate with long, erect hair-like setae; interstrial setae bi- or triseriate with semi-recumbent hair-like setae. Impressed portion of disc covered with leaf-like setae. Declivity sulcate, with a pair of major (largest) spines on declivital summit and four much smaller minor spines on declivital margin, first minor spine located far from the major spine; striae and interstriae punctate, punctures small and shallow; striae 1 shallowly impressed, 2−3 flattened; interstriae 1 slightly convex, 2 and 3 flattened. Striae and interstriae with flattened bristle-like setae, setae semi-recumbent, near median suture in vertical rows (3–4 rows on each side), apically pointed, near lateral margins (four or five rows per side) pointed inwardly to elytral suture, at apical margin without upwardly setae. ***Legs***: procoxae contiguous; prosternal coxal piece short, inconspicuous. Protibiae slender, broadest at middle; posterior face inflated, punctate, densely covered with long hair-like setae; outer margin armed with four or five moderate socketed denticles. Meso- and metatibiae rounded, flat, mesotibiae armed with three or four smaller socketed denticles, metafemora and metatibiae enlarged, the latter without spine.

**Male.** Unknown.

##### Etymology.

L. *inter* + *medius* = in the middle. The name refers to the morphological characters of the species which lie between those of *E.limbus* and *E.spinosus*. An adjective.

##### Distribution.

Thailand (Chiang Mai, Lamphun, Tak, Ubon Ratchathani provinces).

##### Host plants.

Unknown.

### ﻿Key to species *of Eccoptopterus* Motschulsky, 1863 of Indochina (females only)

**Table:** 

1	Declivity bearing one major spine on each elytral margin; declivital armature consisting of two large spines closest to suture on declivital summit and 2–8 minor, uniform-sized denticles on declivital margin	**2**
–	Declivity bearing three major spines on each elytral margin; largest spine near the declivital summit with or without additional 3−4 minor spines between major spines 2 and 3	***Е.spinosus***
2	Declivity with 2–4 minor spines, spines widely separated, unevenly spaced on interstriae; smaller size, 1.70−2.64 mm	**3**
–	Declivity with 6−8 minor spines, spines close together, evenly spaced on each interstriae; larger size, 3.5−4.2 mm	***Е.limbus***
3	Larger body size, 2.56–2.64 mm; outer margins of protibiae with six or seven socketed denticles	***Е.formosanus***
–	Smaller body size, 1.70−1.90 mm, outer margins of protibiae with four or five socketed denticles	***Е.intermedius***

## ﻿Discussion

The differences of both COI and CAD sequences between the new species (*E.formosanus*, *E.intermedius*) and *E.limbus*, *E.spinosus* sensu lato and its junior synonym *E.gracilipes* are clearly greater than suggested species boundaries for these genes (Cognato et al. 2020). The *E.intermedius* specimens demonstrated some DNA sequence differences as illustrated by the branch lengths (Fig. 1). The morphological characters of these geographically separated individuals also exhibited slight variation in the degree of elytral tapering and declivital setal density. These also vary within the type series and are independent of locality and collection date.

The morphological features of *E.intermedius* are more similar to *E.limbus* than to *E.spinosus* but some characters are intermediary (Table 4). *Eccoptopterusintermedius* differs from *E.limbus* by the distinctly smaller size, shorter elytra (elytra: pronotum), the presence of only four minor spines on declivital margin, more slender protibiae. The species differs from *E.spinosus* by hair-like declivital setae and has only a pair of major spines and more slender protibiae.

**Table 4.** Comparative morphological characters for *Eccoptopterus* species.

Species	Total length (mm)	Length/width ratio	Elytral armature on each elytral margin
*E.formosanus*	2.56–2.64	2.13–2.17	1 major on declivital summit, 2–4 minor spines
*E.intermedius*	1.70−1.90	2.03−2.38	1 major on declivital summit, 4 minor spines
*E.limbus*	3.5–4.2	2.1–2.3	1 major on declivital summit, many minor spines
*E.spinosus*	2.5–3.7	2.06–2.27	3 majors on each elytral margin, 0–4 minor spines between major spines 2 and 3

The morphological features of *E.formosanus* are more similar to *E.spinosus* than to *E.limbus*. *Eccoptopterusformosanus* differs from *E.spinosus* by the absence of second and third major spines on declivital margins and declivital face densely covered with thick, long setae. The species differs from *E.limbus* by its distinctly smaller body size, the distinctly tapered elytra, and the declivital margin with 2–4 minor denticles (Table 4).

The two major diagnostic characters used in *Eccoptopterus* species delimitation are the pattern of spines on the declivital margin and the declivital vestiture (Hulcr and Cognato 2013). Hulcr and Cognato (2013) indicated a continuum of morphological variation and geographic origin independence. *Eccoptopterusspinosus* varies greatly in both body size and declivital spine configuration. The configuration of elytral spines in *E.spinosus* and its junior synonym *E.gracilipes* is geographically extremely variable, resulting in inconsistent identifications of *E.spinosus* and *E.gracilipes* in some collections (Hulcr and Cognato 2013). Our study shows non-monophyly and long branch lengths for *E.spinosus* (Fig. 1). The type specimens of *E.spinosus* are presumed lost and the original description is not detailed enough for the comparison of species. *Eccoptopterusspinosus* is likely a species complex, and the combined use of DNA and morphological characters may be the best solution for revising this species and the entire genus (Cognato et al. 2020; Smith et al. 2020).

## Supplementary Material

XML Treatment for Eccoptopterus

XML Treatment for Eccoptopterus formosanus

XML Treatment for Eccoptopterus intermedius

## ﻿Additional information

### Author ORCIDs

Wisut Sittichaya https://orcid.org/0000-0001-6200-1285

Ching-Shan Lin https://orcid.org/0009-0006-3159-697X

Sarah M. Smith https://orcid.org/0000-0002-5173-3736

Chaninan Pornsuriya https://orcid.org/0000-0002-8233-1254

Anthony I. Cognato https://orcid.org/0000-0001-6436-2447

### Data availability

All of the data that support the findings of this study are available in the main text.
